# Application of Craniosacral Therapy Versus Blood Levels of Corticoliberin and Oxytocin in Male Firefighters Exposed to Occupational Stress—A Randomised Control Trial

**DOI:** 10.3390/metabo15060374

**Published:** 2025-06-06

**Authors:** Małgorzata Wójcik, Idzi Siatkowski

**Affiliations:** 1 Department of Physiotherapy, Faculty of Sport Sciences in Gorzow Wielkopolski, Poznan University of Physical Education, 61-871 Gorzów Wielkopolski, Poland; 2 Department of Mathematical and Statistical Methods, Poznan University of Life Science, 60-637 Poznań, Poland; idzi.siatkowski@up.poznan.pl

**Keywords:** stress, firefighters, craniosacral therapy, alternative medicine, corticotrophin-releasing hormone, oxytocin

## Abstract

**Background**: Firefighters’ work exposes them to high levels of stress. Oxytocin (OXT) and corticotrophin-releasing hormone (CRH) are hormones released in response to stress. Prolonged exposure to stress can have negative effects, such as increased blood pressure and glucose levels, and a weakened immune system. **Methods**: This study involved 57 fire department cadets, randomly divided into craniosacral therapy (CS) and contralateral therapy (CO) groups. This study aimed to check whether 5-week craniosacral therapy affects CRH and OXT levels, determined from blood. **Results**: For the CS group, CRH_1 and CRH_2 showed slight increases in median values, 1.73 vs. 2.16, and OXT_1 and OXT_2 showed significant increases in median values, 54.71 vs. 57.77. Spearman’s correlation coefficient for CRH_1 vs. OXT_1 was r = 0.26, *p* = 0.124; similarly, for CRH_2 vs. OXT_2 was r = −0.02, *p* = 0.920; for CRH_ 1 vs. CRH_2 was r = 0.25, *p* = 0.173; and for OXT_1 vs. OXT_2 was r = 0.77, *p* < 0.00001. The values of the point statistics for CRH were similar in CO_1 and CS_1. After the end of therapy, in the CS_2 group, the values of the point statistics were greater than those for the CO_2 group. The median values for oxytocin in the CO_1 group were greater than those in the CS_1 group. After the end of therapy, in the CO_2 group, the values of the scoring statistics were smaller than those for the CS_2 group. The effect of the intervention in the CS group and the CO group showed a significance of *p* = 0.0003 and *p* = 0.023. **Conclusions**: After the end of therapy, a significant increase in OXT levels was observed, as well as a slight increase in CRH levels.

## 1. Introduction

Stress is a common phenomenon that affects people in different ways. It can be caused by various factors such as work, relationships, and health problems. Firefighters and rescue workers are exposed to high levels of stress in their work. They are often called upon to respond to life-threatening emergencies such as fires, natural disasters, and accidents [1]. Physically, they are exposed to hazardous materials, extreme temperatures, and musculoskeletal injuries. Psychologically, they might experience events that can lead to post-traumatic stress disorder, depression, and anxiety [1]. Stress activates the sympathetic nervous system, releasing catecholamines that initiate a coordinated biochemical response, thus leading to an increase in oxytocin (OXT), vasopressin, and corticotropin-releasing hormone (CRH) levels, which facilitate the bio-availability of the hormone adrenocorticotropin. Moreover, stress regulates the hypothalamic–pituitary–adrenal (HPA) axis and the inflammatory response [2].

CRH is a hormone associated with the stress response. When a stressor is triggered, CRH is released in the hypothalamus, frontal cortex, cingulate cortex, central nucleus of the amygdala, locus coeruleus, and raphe nucleus. In a subsequent reaction, CRH stimulates the anterior lobe of the pituitary gland, which secretes corticotropin (ACTH). Corticotropin is transported to the adrenal cortex, from which glucocorticoids, including cortisol (CORT), are secreted [3]. CRH is believed to be a major modulator of the HPA and has an important function in the stress response [3]. These hormones assist the body in coping with stress by raising the heart rate, blood pressure, and blood glucose levels.

Oxytocin is predominantly synthesised in large magnocellular neurons located in the paraventricular and suprachiasmatic nuclei of the hypothalamus. Axons reach the posterior pituitary via the infundibulum, and the peptides are stored in vesicles which release their products into circulation when action potentials reach nerve endings [4].

OXT acts as both a neurohormone and a neuromodulator in many OXT-receptor (OXTR)-expressing brain regions, for instance the hypothalamic nuclei and extrahypothalamic limbic areas [5]. OXTR and OXT synthesis has also been detected in peripheral organs. Oxytocin may play a paracrine role in the periphery. The female reproductive organs, for instance, the fallopian tubes and uterus, contain oxytocin-producing cells, as do the placenta, corpus luteum, epididymis, Leydig cells, prostate, adrenal medulla, reticulum, thymus, and pancreas.

Stress activates the endogenous OXT system [6]. OXT seems to play a role of some importance in human social cognition, motivation, emotion detection, emotion recognition, and emotional memory, in addition to prosocial behaviour, such as trusting behaviour, generosity, and cooperation. OXT has analgesic, anti-anxiety, and anti-stress effects [6]. OXT receptors have been detected on a wide range of immune cell types, including neutrophils, macrophages, and lymphocytes and may be significant in immune surveillance, defence, and homeostasis.

Neumann and Slattery hypothesised that individuals who have higher levels of OXT are less likely to experience stress-related disorders such as depression and anxiety. In stress situations, OXT guarantees the natural mechanism for reducing stress and anxiety [7].

OXT and CRH are inversely correlated. OXT has a calming effect on the body and reduces anxiety, while CRH activates the HPA axis and helps the body cope with stress. OXT inhibits CRH secretion, and this relationship is important for regulating stress responses and maintaining homeostasis in the body. In light of the known pathophysiological mechanisms of oxytocin and corticoliberin for physical and mental health, the role of these neurohormones in promoting resilience to stress requires further study [8].

Short-term stress can be beneficial. It helps people cope with challenging situations and triggers hormonal responses to maintain the body’s homeostasis. This response is commonly known as ‘fight or flight’. However, long-term stress leads to persistently elevated levels of cortisol in the body, which in turn can result in physical and mental health problems like high blood pressure, diabetes, obesity, anxiety, and depression. There is also evidence that chronic stress can have an impact on how nerve cells and synapses function [9]. Chronic stress can also weaken the immune system, increasing susceptibility to infection and disease. Thus, it is important to find ways to manage stress and maintain a healthy balance of these hormones in the body.

Help for the person experiencing excessive anxiety should focus on balancing the autonomic nervous system, lowering the perception of anxiety, and reducing tension in the musculofascial system. Increasingly, attention is being paid to techniques connected with a holistic approach, which entail taking the body and mind into consideration to an equal degree.

The firefighting profession is fraught with disadvantages: high levels of stress [10] and physical, chemical, mechanical, and psychosocial hazards [11]. Individuals who have experienced traumatic events undergo changes in the hypothalamic–pituitary–adrenal (HPA) axis [12], and firefighters are at risk of post-traumatic stress disorder (PTSD) [13]. The profession is also at risk of occupational burnout [14,15,16].

Osteopathy in the cranial sphere was developed in the early 1930s by the osteopathic physicians W. G. Sutherland and C. Weaver. The aim of craniosacral therapy is to alleviate tension and to relax connective tissue structures, whose increased tone is usually considered by osteopathic medicine to be at the root of health problems. Craniosacral therapy is categorised as alternative medicine and is widely used for various dysfunctions. This therapy uses the following approaches: structural, membrane and fluid fluctuations [17].

The effect of this therapy on neuronal pathways is not well understood. The skull is highly innervated and provided with mechanoreceptors. Mechanoreceptors on the other hand, convert mechanical stimuli into electrical and biochemical signals thanks to specialised proteins forming mechanically activated ion channels (MAICs) on the cell membrane [18]. Among the proteins, transient receptor potentials (TRPs) and especially Piezo channels play a very important role [18]. These proteins are involved in neuronal signalling such as temperature regulation, pressure sensing, pH modulation, nociception, and central pain generation [19]. The main influencing factor of craniosacral therapy is the touch of the therapist performing it. The distribution of sensory fibres within the cranium, which is denser in the back of the head and neck (approximately 17 units/cm^2^), can also be significant in the effect of this therapy. C- and Aδ afferent sensory fibres are found in the periosteum of the skull, resulting in sensitivity to mechanical stimulation and nociception [20]. This neural network connects intracranially to the dura mater, influencing its function [20]. The effects of craniosacral therapy on the human body, particularly on the autonomic system, can be explained by the activation of the trigeminal nuclei. This nerve has connections to nuclei in the brainstem and to limbic and cortical structures [21]. Thus, sensory information from the craniofacial nuclei is transmitted to the thalamic nuclei and sensory cortex [21]. The trigeminal sensory nuclei also integrate somatic information from the face into extra-trigeminal structures involved in autonomic and higher functions such as emotional, behavioural, and cognitive functions [22]. The trigeminal nerve system is connected to the rostral ventrolateral medulla (RVLM) in the brainstem and the nuclei of the vagus nerve. In turn, the vagus nerve influences the regulation of autonomic nervous system activity (sympathetic and parasympathetic) at the systemic level [22].

Taking into consideration the beneficial impact of craniosacral therapy and the pathophysiological effects of OXT (increased cognitive performance) and CRH (mobilisation of metabolic resources needed for rapid decision-making during stress), the following research hypotheses were formulated:

1. The application of craniosacral therapy changes the level of CRH;
2. The application of craniosacral therapy changes the level OXT.

The purpose of the statistical analysis was to investigate how craniosacral therapy impacts the interaction between both neuropeptides (CRH and OXT).

In addition, we decided to check whether the following appear in the craniosacral therapy group and control group: relationships between CRH_1 and OXT_1 (the level of corticoliberin versus oxytocin before therapy), between CRH_2 and OXT_2 (the level of corticoliberin versus oxytocin after therapy), between CRH_1 and CRH_2 (before and after), and between OXT_1 and OXT_2 (before and after).

## 2. Materials and Methods

### 2.1. Participants

Fifty-seven firefighter cadets aged 18–24 (21.63 ± 1.41 years), with a mean Body Mass Index of 24.44 ± 3.05 kg/m^2^, volunteered for this study. The sample consisted solely of men, as it has been shown that gender influences circulating cortisol levels (in particular, the phase of the woman’s menstrual cycle). Participants were recruited through meetings organised at the fire academy, posters, and leaflets, and they also obtained comprehensive information about the study project. Those expressing interest in participating in this study were interviewed with regard to the inclusion and exclusion criteria. The inclusion criteria were male gender and being a fire academy cadet, while the exclusion criteria were physiotherapeutic or osteopathic therapy used at present or in the past; daily cigarette smoking; alcohol abuse; caffeine use (>300 mg/day); medication; drug use; reported medical illnesses, including the following types of disorders: endocrine, cardiovascular, and psychiatric diseases. The materials and methods were determined as described previously [23,24,25]. The results presented are for the same group of subjects. This study was carried out during 2017–2018.

### 2.2. Randomisation

Randomisation was determined as described previously [23,24,25].

#### 2.2.1. Corticotropin-Releasing Hormone and Oxytocin Assessment

All blood samples were taken by a qualified nurse in the doctor’s office at the Fire Cadet School after 10–12 h of overnight rest (sleep) at the beginning of this study and at the end of this study, i.e., after five weeks. Blood samples (15 mL) were collected from the basilic vein by a nurse on the morning shift (7:00 a.m. to 9:00 a.m.). Vacutainer tubes were used, into which the cadet’s blood was drawn while in the sitting position. The blood was distributed in one tube with gel and a clot activator (10 mL) to obtain serum. This serum was subsequently stored at −80 °C until analysis. All laboratory measurements were conducted by a medical laboratory scientist. CRH levels were measured using an ELISA Kit for corticotropin-releasing hormone (cat. SEC935Hu) from Cloud Clone Corp. (Katy, TX, USA). All the samples from each part of the experiment (pre/post) were analysed using the same ELISA kit (one kit enables 90 samples to be analysed), the assay sensitivity was 0.8 nmol/L, and the inter- and intraassay variation coefficients were all below 10%. Hormones were determined as described previously [23,24].

#### 2.2.2. Therapeutic Techniques of Craniosacral Therapy

Before performing craniosacral therapy, all participants in the study group (CS—Craniosacral group) (*n* = 30) received an introduction to the therapy. The therapy sessions were held in a warm, quiet room, once a week for five consecutive weeks from 9.30 a.m. to 1 p.m. by the same therapist. Participants lay in a supine position on a couch, with the therapy being conducted on each occasion by the therapist herself, following the established methodology. In this study, we used a structural approach to craniosacral therapy. The therapist applied craniosacral therapy (sacrum compression and traction, AO—Atlanto-occipital joint, mobilisation of the frontal bone, parietal bones, sphenoid bone, and temporal bones), and the final step was the CV4 technique [26]. Participants in the no-intervention group (CO—control group) (*n* = 27) received no therapy. In this group, the therapist only held the subject’s head (while the cadet was in a supinated position) and did not use her hands for any of the techniques. The duration of therapy for individual subjects in both groups was 20 min. None of the 57 subjects had received osteopathic or physiotherapeutic therapy prior to this study, and the subjects had no previous knowledge or experience of osteopathic craniosacral treatments.

#### 2.2.3. Statistical Analysis

The statistical analysis was performed using R ver. 4.2.2 software [27]. Both the craniosacral therapy experimental group and the control group were analysed. As the data do not follow a normal distribution (the Shapiro–Wilk test was used to check the normality distribution), the paired non-parametric Wilcoxon test was used. The values of the CRH and OXT levels measured at two time points (before and after intervention) were subjected to a comparison for the experimental and control groups. However, the simultaneous effect of the groups on the levels of these hormones was analysed using a linear model. Spearman’s correlation testing was performed when analysing the effects of CRH and OXT in the craniosacral therapy group and the control group without intervention.

## 3. Results

### 3.1. Craniosacral Therapy Group

#### 3.1.1. Corticotropin-Releasing Hormone

**Figure 1:**
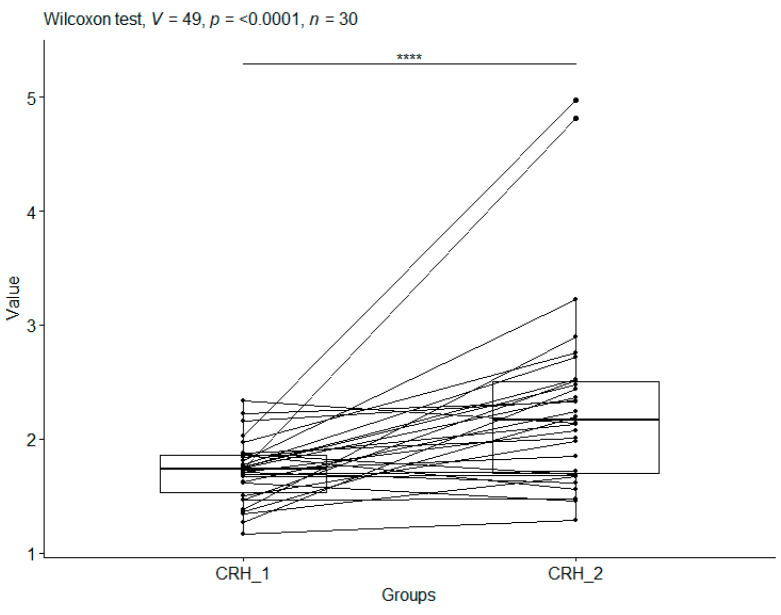
CRH ng/mL for the CS group. Boxplots show, for the same individual, CRH values before therapy (CRH_1) and CRH values after therapy (CRH_2). In addition, boxplots were made for CRH_1 and CRH_2. Significant codes—****: *p*-value < 0.0001.

After applying the Wilcoxon test to compare CRH_1 and CRH_2 in the CS group (median values: 1.73 vs. 2.16—a slight increase), a *p*-value < 0.0001 was obtained. This means that craniosacral therapy had a statistically significant effect on corticoliberin levels (Figure 1).

#### 3.1.2. Oxytocin

**Figure 2:**
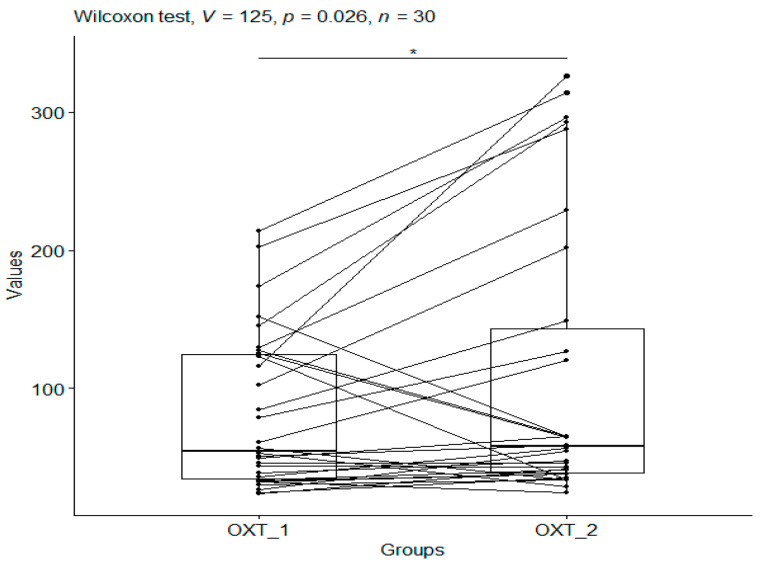
OXT pg/mL for the CS group. The boxplots show CRH values before therapy (CRH_1) and CRH values after therapy (CRH_2) for the same individual. In addition, boxplots were produced for OXT_1 and OXT_2. Significant codes—*: *p*-value 0.01 < *p* < 0.05.

After applying the Wilcoxon test to pool the data comparing OXT_1 with OXT_2 in the CS group (median values: 54.71 vs. 57.77—a significant increase), a *p*-value of 0.026 was obtained, indicating that craniosacral therapy had a statistically significant effect on oxytocin levels (Figure 2).

### 3.2. Spearman’s Correlations

The next step in the statistical analysis was to establish Spearman’s correlations and investigate the relationship between CRH (corticoliberin) and OXT (oxytocin) levels in the CS group before and after therapy.

For CRH_1 vs. OXT_1 (corticoliberin and oxytocin before therapy), the Spearman’s correlation coefficient value was r = 0.26, with a *p*-value = 0.124 (Table 1), indicating no significant correlation between the levels of these hormones before therapy. Similarly, no significant correlation between hormone levels was observed in the group after craniosacral therapy (the Spearman’s correlation coefficient for CRH_2 vs. OXT_2 was r = −0.02, with a *p*-value = 0.920; Table 1), suggesting these variables were not statistically dependent.

Comparing corticoliberin prior to (CRH_1) and after therapy (CRH_2), a Spearman’s correlation coefficient of r = 0.25 was obtained, indicating a weak positive correlation. However, with a *p*-value = 0.173, there is no significance in this correlation (Table 1), suggesting the analysed characteristics are not statistically dependent.

In contrast, comparing oxytocin before therapy (OXT_1) and after therapy (OXT_2), a Spearman’s correlation coefficient of r = 0.77 with a *p*-value < 0.00001 was observed, indicating a significant and strong positive relationship (Table 1). Therefore, there is a significant correlation for oxytocin levels before and after therapy, meaning that oxytocin levels changed significantly after therapy.

### 3.3. Control Group (Head Holding)

In addition, in the control group, the condition of satisfying the normal distribution of the data was checked with the Shapiro–Wilk normality test before commencing statistical analysis. In this group, the data did not follow a normal distribution either. The Wilcoxon test for data coupling was applied.

### 3.4. Corticotropin-Releasing Hormone

**Figure 3:**
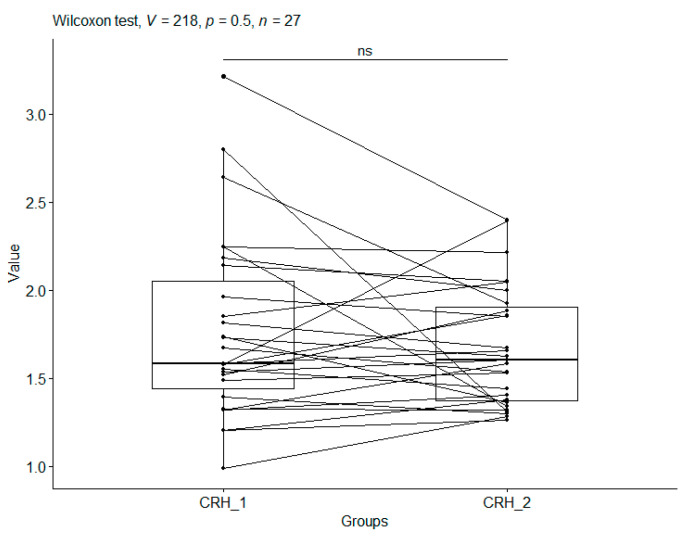
Corticoliberin (CRH ng/mL) for the control group (CO). Boxplots for the same individual show CRH values before the start of this study (CRH_1) and CRH values after the end of this study (CRH_2). In addition, boxplots were produced for CRH_1 and CRH_2. Significant codes—ns: nonsignificant.

After applying the Wilcoxon test for data coupling to compare CRH_1 with CRH_2 in the CO group (median values: 1.58 vs. 1.60 ng/mL), a *p*-value (*p* = 0.499) was obtained. This meant that the values for CRH_1 (before therapy) and CRH_2 (after therapy) for this group are not significantly different. In view of this, we may conclude that no effect of the head holding on CRH (corticoliberin) values was noted in the control group (Table 2, Figure 3).

### 3.5. Oxytocin

**Figure 4:**
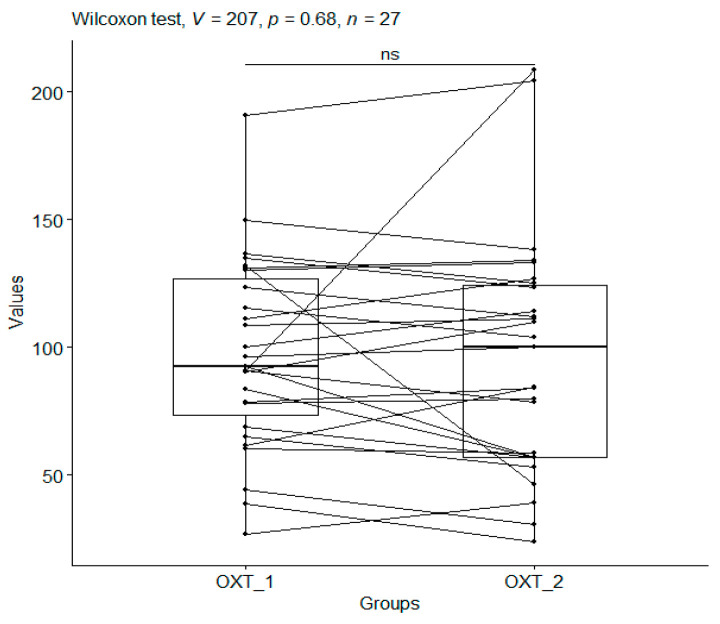
Oxytocin (OXT pg/mL) for the control group (CO). The sections show, for the same individual, the OXT values before (OXT_1) and after this study (OXT_2). In addition, boxplots were produced for OXT_1 and OXT_2. Significant codes—ns: nonsignificant.

A *p*-value = 0.679 obtained from the Wilcoxon test when comparing oxytocin in the CO group before and after the study (median values: 92.55 vs. 99.96 pg/mL) does not indicate that holding the head influenced oxytocin levels (Table 3, Figure 4). There was no significant reduction in oxytocin levels after this study.

### 3.6. Spearman’s Correlation

After performing the correlation tests, no relationship was noted between corticoliberin (CRH_1) and oxytocin (OXT_1) prior to the start of this study in the control group (r = 0.20, *p*-value = 0.300) (Table 1) and after it ended (r = 0.14, *p*-value = 0.469) (Table 1). These findings show that head holding had no impact on corticoliberin and oxytocin levels. This in turn implies the lack of a statistical correlation between hormone levels before the research.

Statistical significance was found for corticoliberin before and after the study for CRH_1 vs. CRH_2 (r = 0.51, *p*-value = 0.006) (Table 1). Strong significant correlation was observed for OXT_1 vs. OXT_2 (r = 0.73, *p*-value < 0.00001) (Table 1). A correlation was observed for oxytocin before and after the start of this study, indicating a decrease.

#### Treatment Group vs. Control Group

The basic statistical characteristics for CRH in the craniosacral therapy group and control group are presented in Table 2.

Analysing Table 2 and Table 3, it should be noted that before the start of therapy, in both study groups (CO_1 and CS_1), the values of the point statistics for CRH are similar. On the other hand, after the end of therapy, in the CS_2 group, the values of the point statistics are greater than those for the CO_2 group.

The basic statistical characteristics for oxytocin in the treatment and control groups are shown in Table 3.

Analysing Table 3, it should be noted that before the start of therapy, the median values (mean and median = Q50) for oxytocin in the CO_1 group were greater than those in the CS_1 group. On the other hand, after the end of therapy, in the CO_2 group, the values of the scoring statistics were smaller than those for the CS_2 group.

The next step in the statistical comparison between the effect of the intervention in the CS treatment group and the CO control group was to use the following procedure: the differences for CRH/OXT before and after treatment in the CS and CO groups were calculated, and then, the CS group was compared with the CO group for the resulting data. To this end, it was first verified whether there was a normal distribution using the Shapiro–Wilk normality test. A *p*-value= 0.0001 was obtained for CRH, and a *p*-value = 0.0048 for CO, indicating that the data do not follow a normal distribution. Therefore, when comparing the CS vs. CO groups, the Wilcoxon test was applied and a *p*-value = 0.0003 was obtained. This means that the CS and CO groups are statistically significantly different for CRH, i.e., differences in CRH values after therapy and before therapy.

A further analysis was conducted to check the therapeutic effect for the hormone oxytocin. Also, the Shapiro–Wilk normality test was applied. The *p*-values obtained for the CS group were 0.1394 and for the CO group < 0.0001.

Since the data for the CO group do not follow a normal distribution, when comparing oxytocin in the CS vs. CO group, the Wilcoxon test was applied and a *p*-value = 0.023 was obtained. This means that the CS and CO groups are statistically significantly different for oxytocin, i.e., differences in OXT values after therapy and before therapy.

## 4. Discussion

The results obtained show a significant increase in OXT and a slight increase in CRH after craniosacral therapy. Oxytocin may inhibit CRH release, which induces cortisol release during the stress response. Thus, oxytocin has a modulatory role within the HPA axis [28,29]. Oxytocin may influence the cognitive and social consequences of stress by acting on prefrontal cortex (mPFC) circuits to coordinate responses to OXT and CRH [30]. OXT mRNA expression has been shown to increase in PVN and multicellular SON neurons during chronic stress [28]. While OXT is mainly involved in performing social behaviour, CRH is largely involved in regulating stress and anxiety [29]. Animal models and human studies have shown that OXT has anti-anxiety effects and CRH induces depression [31]. Stress-related overactive OXT–CRH brain systems can induce major depressive disorder and bipolar disorder [31]. The firefighting profession is particularly vulnerable to high levels of anxiety and traumatic stress, resulting from participation in incidents [1]. Perhaps the resulting increase in CRH is due to being in the same environment all the time (the study participants were in the barracks) and to activities resulting from firefighter preparation training, e.g., taking part in firefighting operations and accidents. Participants in our study had to be constantly ready for possible participation in an occurrence. Unfortunately, it was not possible for our research participants to be excluded from their duties for the duration of this study.

In the literature, there is a lack of references to studies using craniosacral therapy in which the therapy was used as a means to affect CRH and OXT hormones. To date, our own research has revealed how this therapy lowers cortisol [23,24]. On the other hand, this therapy has been shown to have a positive effect on heart rate variability (HRV) [25].

A study by Zhang et al. used vibratory abdominal massage in insomniacs to show how the treatment affects the HPA axis (hypothalamic–pituitary–adrenal), affecting CRH production levels, and improves sleep [32]. Zhu et al.’s study on mice showed that electroacupuncture affects the reduction in CRH levels [33]. On the other hand, the methodology of performing a massage or electroacupuncture and craniosacral therapy differs radically. CRH is the origin and main driver of the HPA axis, whose neurons are located in the paraventricular nucleus (PVN) of the hypothalamus [34]. This guarantees the body has the appropriate stress responses and maintains a heightened state of alertness. In stress regulation, a mutual impact of OXT and the CRH system can be observed. In response to stress, CRH neurons release CRH peptides into the median suculus. This, in turn, stimulates the release of pituitary adrenocorticotrophic hormone (ACTH) and, ultimately, the adrenal hormone [35]. On the other hand, OXT affects the regulation of the HPA axis through negative feedback by glucocorticoids (GCs). OXT directly and immediately produces an amplifying effect on ACTH secretion, which is induced by CRH [35].

In this study, a strong correlation was also observed in both the CS and CO groups for OXT level values before and after the study. There is no reference in the literature to studies in which CS therapy was used to check its effect on OXT. However, 15 min of moderate-pressure massage was applied to the upper back, which was found to have an effect of increasing OXT and decreasing ACTH, nitric oxide (NO), and beta-endorphin (BE) [36]. The use of acupuncture can also directly up-regulate the level of OXT in the hypothalamus [37]. On the other hand, as previously mentioned, the methodology of massage and acupuncture differs significantly from CS therapy. Mehta et al. observed that performing yoga can also increase OXT in patients with psychiatric illnesses [38].

OXT regulates not only the brain and the reproductive system but also the immune system and the autonomic nervus system (ANS), involving changes in states and emotional regulation. It is already known that the main neurotransmitters of the parasympathetic nervous system, i.e., acetylcholine and oxytocin, originate from colocalised molecules and their receptors in tissues, including areas of the hypothalamus and brain stem that regulate ANS [39]. Acting through the ANS on the cerebral cortex and brain stem, OXT is involved in human social reactions, i.e., the expression of emotions on the face and through gaze, and also influences the capability and expression of social bonds that are formed in response to experiences or failures [40]. Animal model studies suggest the interdependence of OXT and gamma-aminobutyric acid (GABA) in regulating social behaviour [40]. OXT also has a protective function for nerve cells against hypoxia and ischaemia by preserving mitochondrial function; reducing oxidative stress; and decreasing chromatin protein, which is released during inflammation. The mechanisms through which OXT attenuates central stress responses, specifically its actions on CRH neurones, have yet to be elucidated. However, recent evidence suggests that very few parvocellular CRH neurones express an mRNA transcript for the OXT receptor postsynaptically [41].

Anxiety disorders are a group of common disorders which can have a debilitating effect on a person’s daily functioning and well-being. The most important neuropeptides that play a role in modulating stress and anxiety-related behaviours are cholecystokinin, oxytocin, and ghrelin [42]. OXT plays a role in the pathophysiology of anxiety disorders. These disorders may co-occur with other psychiatric disorders, such as depression, and may predispose to the risk of cardiovascular disease and premature mortality. Persistent anxiety and the chronic stress associated with it induce pro-inflammatory changes which are directly linked to the hypothalamic–pituitary axis (HPA), thereby increasing the risk of excessive systemic inflammation [43].

Kenel et al. demonstrated that gender differences in the oxytocin–vasopressin system are highly noticeable in the presence of stressful experiences, including social and hormonal experiences in early life [44]. Oxytocin secretion may be involved in the self-regulatory process that helps to cope with stress and recovery from trauma [45]. OXT and CORT (cortisol) are included among the potential markers of anxiety disorders.

The research results presented are promising and point to the use of craniosacral therapy as one way of dealing with stress.

## 5. Conclusions

After the end of therapy, a significant increase in OXT levels was observed, as well as a slight increase in CRH levels. The results show that this therapy can be a way to reduce stress levels in firefighters and in people exposed to high levels of stress. Perhaps craniosacral therapy should be performed more than once a week, for longer than 20 min, and for a longer period of time than 5 weeks.

### Limitations

Several limitations of this study should be noted. Firstly, only men participated in this study. This type of study should be conducted with women both using and not using hormonal therapy (contraceptive therapy and menopausal therapy) and with participants from different age and social groups. Secondly, our sample of 57 men was small—only 30 in the study group and 27 in the control group. Therefore, these results should be regarded as preliminary, pending further research.

## Figures and Tables

**Table 1:** Spearman’s correlations, r—correlation coefficient, *p*-value determined for the correlation coefficient test. CS—craniosacral therapy group; CO—placebo control group; CRH_1 vs. OXT_1—corticoliberin and oxytocin before craniosacral therapy; CRH_2 vs. OXT_2—corticoliberin and oxytocin after craniosacral therapy; CRH_1 vs. CRH_2—corticoliberin before and after craniosacral therapy; OXT_1 vs. OXT_2—oxytocin before and after craniosacral therapy.

Comparison	r for CS	*p*-Value for CS	r for CO	*p*-Value for CO
CRH_1 vs. OXT_1	0.26	0.124	0.20	0.300
CRH_2 vs. OXT_2	−0.02	0.920	0.14	0.469
CRH_1 vs. CRH_2	0.25	0.173	0.51	0.006
OXT_1 vs. OXT_2	0.77	<0.00001	0.73	<0.00001

**Table 2:** Serum concentration of corticoliberin (ng/mL) in the craniosacral therapy group and control group (CS—craniosacral therapy group; CO—control group; 1—corticoliberin, with values before therapy in the CS group and in the CO group before the start of this study, and 2—after therapy in the CS group and in the CO group after the end of this study; mean—the arithmetic mean, std—standard deviations, n—sample size, min—minimum, max—maximum, Q25—first quartile, Q50—second quartile = median, Q75—third quartile).

Group	Mean	std	n	Min	Max	Q25	Q50	Q75
CO_1	1.77	0.52	27	0.98	3.21	1.43	1.58	2.04
CO_2	1.67	0.34	27	1.26	2.39	1.36	1.60	1.90
CS_1	1.71	0.27	30	1.16	2.33	1.53	1.73	1.85
CS_2	2.30	0.84	30	1.28	4.97	1.70	2.16	2.50

**Table 3:** Serum concentration of oxytocin (pg/mL) in the craniosacral therapy group and control group (CS—craniosacral therapy group; CO—control group; 1—corticoliberin, with values before therapy in the CS group and in the CO group before the start of this study, and 2—after therapy in the CS group and in the CO group after the end of this study; mean—the arithmetic mean, std—standard deviations, n—sample size, min—minimum, max—maximum, Q25—first quartile, Q50—second quartile = median, Q75—third quartile).

Group	Mean	std	n	Min	Max	Q25	Q50	Q75
CO_1	97.34	37.17	27	26.76	190.66	73.34	92.55	126.67
CO_2	96.00	46.61	27	23.79	208.62	56.88	99.96	124.03
CS_1	81.60	56.19	30	23.79	214.08	34.41	54.71	124.49
CS_2	108.65	101.42	30	24.71	326.05	38.79	57.77	143.52

## Data Availability

The data are available upon request.

## Abbreviations

The following abbreviations are used in this manuscript:

CS	Craniosacral therapy group
CO	Control group (head holding)
CRH	Corticotropin-releasing hormone
OXT	Oxytocin
1	First measurement before the start of this study in both groups
2	Second measurement after this study in both groups

## References

[1] Lebeaut A., Tran J.K., Vujanovic A.A. (2020). Posttraumatic stress, alcohol use severity, and alcohol use motives among firefighters: The role of anxiety sensitivity. Addict. Behav..

[2] Furman D., Campisi J., Verdin E., Carrera-Bastos P., Targ S., Franceschi C., Ferrucci L., Gilroy D.W., Fasano A., Miller G.W. (2019). Chronic inflammation in the etiology of disease across the life span. Nat. Med..

[3] Horváth K., Juhász B., Kuti D., Ferenczi S., Kovács K.J. (2023). Recruitment of Corticotropin-Releasing Hormone (CRH) Neurons in Categorically Distinct Stress Reactions in the Mouse Brain. Int. J. Mol. Sci..

[4] Cilz N.I., Cymerblit-Sabba A., Young W.S. (2019). Oxytocin and vasopressin in the rodent hippocampus. Genes Brain Behav..

[5] Jiang J., Yang M., Tian M., Chen Z., Xiao L., Gong Y. (2023). Intertwined associations between oxytocin, immune system and major depressive disorder. Biomed. Pharmacother..

[6] Goetz L., Jarvers I., Schleicher D., Mikan K., Brunner R., Kandsperger S. (2021). The role of the endogenous oxytocin system under psychosocial stress conditions in adolescents sufering from anxiety disorder: Study protocol for a parallel group con-trolled trial. BMC Psychol..

[7] Neumann I.D., Slattery D.A. (2016). Oxytocin in general anxiety and social fear: A translational approach. Biol. Psychiatry.

[8] Gutkowska J., Jankowski M., Antunes-Rodrigues J. (2014). The role of oxytocin in cardiovascular regulation. Braz. J. Med. Biol. Res..

[9] Denkova E., Zanesco A.P., Rogers S.L., Jha A.P. (2020). Is resilience trainable? An initial study comparing mindfulness and relaxa-tion training in firefighters. Psychiatry Res..

[10] Sawhney G., Jennings K.S., Britt T.W., Sliter M.T. (2018). Occupational stress and mental health symptoms: Examining the moderating effect of work recovery strategies in firefighters. J. Occup. Health Psychol..

[11] Cuenca-Lozano M.F., Ramírez-García C.O. (2023). Occupational Hazards in Firefighting: Systematic Literature Review. Saf. Health Work.

[12] Pineles S.L., Rasmusson A.M., Yehuda R., Lasko N.B., Macklin M.L., Pitman R.K., Orr S.P. (2013). Predicting emotional responses to potentially traumatic events from pre-exposure waking cortisol levels: A longitudinal study of police and firefighters. Anxiety Stress Coping.

[13] Xue C., Ge Y., Tang B., Liu Y., Kang P., Wang M., Zhang L. (2015). A meta-analysis of risk factors for combat-related PTSD among military personnel and veterans. PLoS ONE.

[14] Makara-Studzińska M., Golonka K., Izydorczyk B. (2019). Self-Efficacy as a Moderator between Stress and Professional Burnout in Firefighters. Int. J. Environ. Res. Public Health.

[15] Vaulerin J., Colson S.S., Emile M., Scoffier-Mériaux S., d’Arripe-Longueville F. (2016). The Big Five Personality Traits and French Firefighter Burnout: The Mediating Role of Achievement Goals. J. Occup. Environ. Med..

[16] Tao Y., Ma Z., Hou W., Zhu Y., Zhang L., Li C., Shi C. (2022). Neuroticism Trait and Mental Health Among Chinese Firefighters: The Moderating Role of Perceived Organizational Support and the Mediating Role of Burnout—A Path Analysis. Front. Public Health.

[17] Wójcik M., Placek K., Bordoni B. (2023). Application of craniosacral therapy in practice. Fizjoterapia Pol..

[18] Coste B., Mathur J., Schmidt M., Earley T.J., Ranade S., Petrus M.J., Dubin A.E., Patapoutian A. (2010). Piezo1 and Piezo2 are Essential Components of Distinct Mechanically Activated Cation Channels. Science.

[19] Muller C., Morales P., Reggio P.H. (2019). Cannabinoid Ligands Targeting TRP Channels. Front. Mol. Neurosci..

[20] Schueler M., Messlinger K., Dux M., Neuhuber W.L., De R. (2013). Extracranial Projections of Meningeal Afferents and Their Impact on Meningeal Nociception and Headache. Pain.

[21] Terrier L.M., Hadjikhani N., Destrieux C. (2022). The Trigeminal Pathways. J. Neurol..

[22] Panneton W.M., Gan Q. (2020). The Mammalian Diving Response: Inroads to Its Neural Control. Front. Neurosci..

[23] Wójcik M., Siatkowski I., Żekanowska E. (2022). A proposal for the use of craniosacral therapy in firefighter cadets to decrease cortisol levels and improve postural stability. J. Men’s Health.

[24] Wójcik M., Bordoni B., Siatkowski I., Żekanowska E. (2023). The Effect of Craniosacral Therapy on Blood Levels of Stress Hormones in Male Firefighter Cadets: A Randomized Clinical Trial. Behav. Sci..

[25] Wójcik M., Siatkowski I. (2023). The efect of cranial techniques on the heart rate variability response to psychological stress test in frefghter cadets. Sci. Rep..

[26] Liem T. (2022). Praktyka Osteopatii Czaszkowo-Krzyżowej. Wrocław.

[27] Development Core Team (2020). A Language and Environment for Statistical Computing.

[28] Gołyszny M. (2018). An “old” and “new” neuropeptides as modulators of the stress axis (hypothalamus–pituitary–adrenal). Varia Medica.

[29] Wei F., Xian D., He Y., Yan Z., Deng X., Chen Y., Zhao L., Zhang Y., Li W., Ma B. (2022). Effects of maternal deprivation and environmental enrichment on anxiety-like and depression-like behaviors correlate with oxytocin system and CRH level in the medial-lateral habenula. Peptides.

[30] Hurlemann R., Marsh N. (2017). Deciphering the modulatory role of oxytocin in human altruism. Rev. Neurosci..

[31] Guo L., Qi Y.J., Tan H., Dai D., Balesar R., Sluiter A., van Heerikhuize J., Hu S.H., Swaab D.F., Bao A.M. (2022). Different oxytocin and corticotropin-releasing hormone system changes in bipolar disorder and major depressive disorder patients. EBioMedicine.

[32] Zhang Y., Cong D., Liu P., Zhi X.Y., Shi C., Zhao J., Zhang H. (2021). Study on the mechanism of regulating the hypothalamic cortical hormone releasing hormone/corticotropin releasing hormone type I receptor pathway by vibro-annular abdominal massage under the brain-intestine interaction in the treatment of insomnia. Medicine.

[33] Zhu J., Wang C., Wang Y., Guo C., Lu P., Mou F., Shao S. (2022). Electroacupuncture alleviates anxiety and modulates amygdala CRH/CRHR1 signaling in single prolonged stress mice. Acupunct. Med..

[34] Jamieson B.B., Nair B.B., Iremonger K.J. (2017). Regulation of hypothalamic corticotropin-releasing hormone neurone excitability by oxytocin. J. Neuroendocrinol..

[35] Pati D., Krause E.G., Frazier C.J. (2022). Intrahypothalamic effects of oxytocin on PVN CRH neurons in response to acute stress. Curr. Opin. Endocr. Metab. Res..

[36] Schneider E., Hopf D., Aguilar-Raab C., Scheele D., Neubauer A.B., Sailer U., Hurlemann R., Eckstein M., Ditzen B. (2023). Affectionate touch and diurnal oxytocin levels: An ecological momentary assessment study. Elife.

[37] Su T., Pei L. (2021). Acupuncture and oxytocinergic system: The promising treatment for autism. Transl. Neurosci..

[38] Mehta U.M., Gangadhar B.N. (2019). Yoga: Balancing the excitation-inhibition equilibrium in psychiatric disorders. Prog. Brain Res..

[39] Quintana D.S., Rokicki J., van der Meer D., Alnæs D., Kaufmann T., Córdova-Palomera A., Dieset I., Andreassen O.A., Westlye L.T. (2019). Oxytocin pathway gene networks in the human brain. Nat. Commun..

[40] Danoff J.S., Whelan E.A., Connelly J.J. (2023). Is oxytocin receptor signaling really dispensable for social attachment?. Compr. Psychoneuroendocrinol..

[41] Chen R., Wu X., Jiang L., Zhang Y. (2017). Single-cell RNA-seq reveals hypothalamic cell diversity. Cell Rep..

[42] Gupta D., Chuang J.C., Mani B.K., Shankar K., Rodriguez J.A., Osborne-Lawrence S., Metzger N.P., Zigman J.M. (2019). β1-adrenergic receptors mediate plasma acyl-ghrelin elevation and depressive-like behavior induced by chronic psychosocial stress. Neuropsychopharmacology.

[43] Svingen S. (2023). PTSD and crime propensity: Stress systems, brain structures, and the nature of the relationship. Heliyon.

[44] Kenkel W.M., Perkeybile A.M., Yee J.R., Pournajafi-Nazarloo H., Lillard T.S., Ferguson E.F., Wroblewski K.L., Ferris C.F., Carter C.S., Connelly J.J. (2019). Behavioral and epigenetic consequences of oxytocin treatment at birth. Sci. Adv..

[45] Carter C.S., Kenkel W.M., MacLean E.L., Wilson S.R., Perkeybile A.M., Yee J.R., Ferris C.F., Nazarloo H.P., Porges S.W., Davis J.M. (2020). Is Oxytocin “Nature’s Medicine”?. Pharmacol. Rev..

